# Pilot testing of an online training module about screening for acute HIV infection in adult patients seeking urgent healthcare

**DOI:** 10.1093/inthealth/ihy077

**Published:** 2018-11-02

**Authors:** Hannah Rafferty, Oscar Chirro, Clifford Oduor, Elizabeth Wahome, Caroline Ngoi, Elise van der Elst, René Berger, Sarah Rowland-Jones, Susan M Graham, Eduard J Sanders

**Affiliations:** 1 KEMRI/Wellcome Trust Research Programme Centre for Geographic Medicine Research—Coast, Kilifi, Kenya; 2 United States Agency for International Development (USAID), Nairobi, Kenya; 3 Nuffield Department of Medicine, University of Oxford, Headington, Oxford, UK; 4 University of Washington, 359909, 325 Ninth Avenue, Seattle, WA, USA

**Keywords:** acute HIV infection, febrile patient, online learning, screening algorithm, self-directed learning

## Abstract

**Background:**

Acute HIV infection (AHI) is the phase of HIV infection immediately after acquisition, during which many patients develop symptoms and often seek healthcare. However, clinicians in sub-Saharan Africa are not currently taught about AHI.

**Methods:**

This study pilot-tested a self-directed AHI training module among clinical officers (COs) in coastal Kenya and assessed knowledge gained and challenges to instituting screening. The training module included four domains: AHI definition and importance of AHI recognition; symptoms and screening algorithms; diagnostic strategies; and management. AHI knowledge was assessed before and immediately after training. Participants’ ability to utilize an AHI screening algorithm was evaluated with a case-based exercise.

**Results:**

Self-directed training was completed by 45 COs. Pre-test scores were low (median score 35% IQR 30–45%), but improved significantly after training (median post-test score 75%, IQR 70–85%, Wilcoxon signed-rank test p<0.0001). Participants had challenges in understanding the utility and application of a screening algorithm to identify patients for whom AHI testing would be indicated. Knowledge of AHI was poor at baseline, but improved with self-directed learning. Based on these findings, we revised and improved the AHI training module and pre- and post-assessments, which are now freely available online at www.marps-africa.org.

**Conclusions:**

Guidelines on AHI screening and diagnosis are urgently needed in high HIV transmission areas.

## Introduction

Early identification and prompt treatment of adults newly infected with HIV-1 is vitally important, both to prevent onward HIV-1 transmission and for the long-term health of the infected individual. Identifying adults with acute HIV-1 infection (AHI), of whom a substantial portion may seek urgent care,^1,2^ is therefore a matter of public health importance. Increasingly, AHI testing has been a focus of research and programmes in well-resourced settings,^3–6^ and has been recommended in several guidelines.^7–9^ Unfortunately, there has been a lack of emphasis on this strategy in resource-limited settings, including sub-Saharan Africa (sSA), where the epidemic has had the greatest impact.^10,11^

AHI is the phase of HIV-1 infection immediately after acquisition, and is characterized by a burst of viraemia, during which 40–90% of patients develop symptoms.^7^ During this time, anti-HIV antibodies are undetectable, but HIV RNA and p24 antigen are present. Once HIV antibodies have become detectable, the phase is usually referred to as early HIV infection (EHI), which corresponds to the first 6 months of infection after acquisition.^12^ Symptoms usually develop around 2 weeks after HIV-1 acquisition, just preceding the peak in viral load.^13,14^ The proportion of AHI patients with symptoms who seek care may range from 29 to 69%.^1,2^ The number of symptoms correlates with higher pre-seroconversion peak plasma viral load.^15^ Thus, strategies to target AHI testing to symptomatic patients at risk for acute and early HIV (AEHI) may identify persons with higher peak viral loads^15^ and higher viral load set points.^13,16^ These patients may be at greatest risk of onward transmission and are, therefore, a priority for screening and early treatment.^17^

A recent scoping review on clinical and public health implications of AEHI detection and treatment identified implementation research as a critical enabler to facilitate sustainable integration of AHI detection and treatment into existing health systems.^12^ There is a paucity of research evaluating HIV education for health professionals, especially those working in sSA. The authors were unable to find any publications specifically concerning AHI education or training for healthcare professionals in this region. Available guidelines for the management of adult outpatients presenting with fever are heavily focused on diagnosing malaria and poorly defined in terms of evaluating other aetiologies.^11^

Here, the pilot testing of an AHI training module developed specifically for primary care clinicians in Kenya is reported. The authors delivered a self-directed educational module about AHI to in-service and pre-service clinical officers (COs) in coastal Kenya. The primary objective of the study was to assess knowledge gained and areas for improvement of the module. Secondary objectives were to assess potential for online provision of both the training module and training COs in the use of a screening algorithm to identify young, at-risk adults who should be tested for AEHI.

## Materials and methods

### Developing the module

A concise self-directed training module was developed to encompass four main domains:

- AHI definition and importance of AHI recognition;
- diagnostic strategies;
- symptoms and screening algorithm;
- management.

Recent AHI literature relevant to each section, as well as an available AHI training module from Australia,^18^ were reviewed. The authors were unable to find any other AHI training modules during the literature search. The module was reviewed by a clinician epidemiologist (SMG) and public health physician (EJS), both with over 10 years of experience with AHI screening in East Africa.

#### Domain 1: AHI definition and importance of recognition

This domain included a definition of AHI as the period immediately after HIV acquisition, characterized by a burst of viraemia, during which 40–90% of patients develop symptoms, and HIV-antibodies are not yet detected.^7^ This domain also explained the importance of AHI diagnosis—to provide treatment without delay and to reduce transmission via treatment for prevention, and effective risk reduction counselling. Since 2016, Kenya has adopted the World Health Organization (WHO) recommendation to treat all HIV-infected persons immediately, irrespective of CD4 count test result.^19^ The five multiple choice questions for this domain focused on:

- the definition of AHI;
- the definition of prevalent HIV;
- the infectivity of AHI;
- the importance of AHI diagnosis;
- the reason why earlier treatment is better for HIV-infected patients.

#### Domain 2: diagnostic strategies

This domain explained which tests could be used to diagnose AHI and when these become positive. To illustrate when each of these tests becomes positive, a graph was provided with HIV viral load vs days following infection and when each test becomes positive. The five multiple choice questions for this domain focused on:

- the change in viral load during AHI;
- how to diagnose AHI;
- the first test to become positive after HIV acquisition;
- the time delay before the rapid antibody HIV test will be positive;
- the relevance of discordant rapid test results (one rapid test positive one rapid test negative).

#### Domain 3: symptoms and screening algorithm

This domain included the prevalence of different symptoms experienced during AHI and when these tend to develop. It introduced the screening score and gave an explanation as to how this score was derived in a study of adults aged 18–35 years.^17^ The five multiple choice questions asked:

- how common it was for AHI patients to have symptoms;
- which symptoms were commonly experienced;
- when these symptoms developed;
- why a screening algorithm may be helpful to a clinician.
- to choose which one of four patients should be tested for AHI, given their ages and symptoms.

#### Domain 4: management

**Figure 1.**
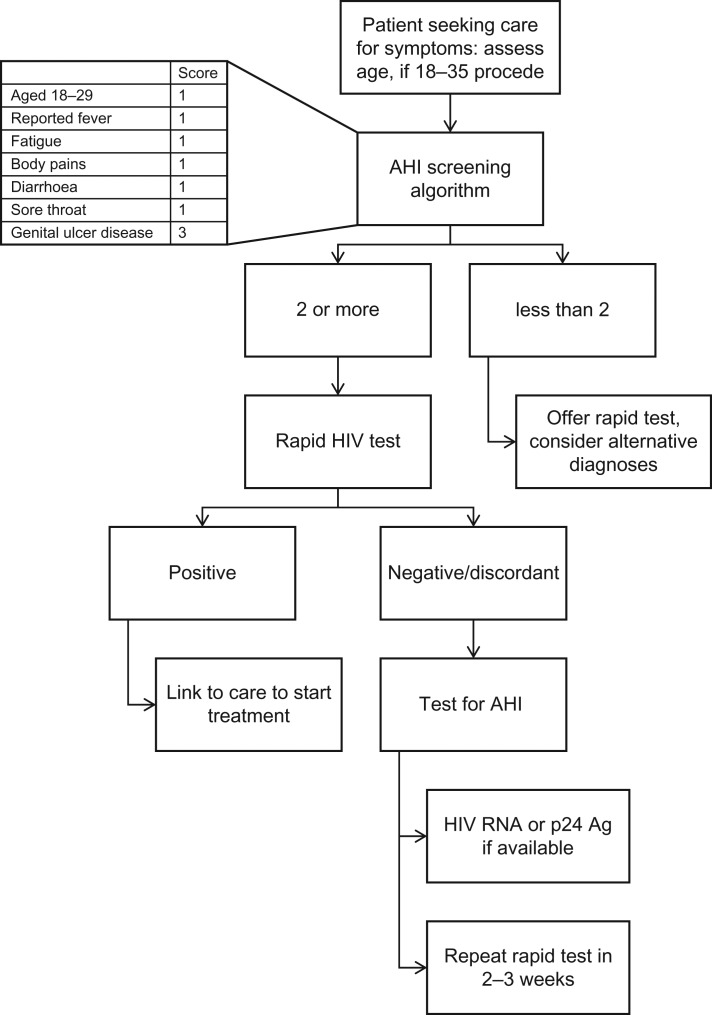
Flow chart for management of patient aged 18–35 seeking care for symptoms.

As there are no current guidelines for testing or management of AHI in sSA, a flow diagram for the management of AHI was developed, which was included in the recently published scoping review supported by authors from WHO^12^ (Figure 1). The five multiple choice questions examined:

- participant knowledge about the lack of AHI guidelines from the WHO;
- current WHO guidelines for anti-retroviral therapy (ART) initiation adopted in Kenya;^19^
- potential treatments for AHI;
- the time interval required for a repeat rapid HIV test;
- the topics that should be discussed with a patient during counselling before and after AHI testing.

COs participating in the study were provided with a printed booklet in English of approximately 3400 words, including three figures over 12 A4 pages (Supplementary information). The module was revised for the second training session after difficulties in comprehension of the screening algorithm. A paragraph was added explaining the age limits of the algorithm and why younger patients score an extra point.

### AHI screening algorithm used in the training module

In the past 15 years, there have been four screening algorithms developed for AHI testing in sSA by research groups in Kenya, Malawi and South Africa.^17^ These algorithms require scoring of sociodemographic factors, sexual risk behaviour, signs and symptoms, and discordant HIV test results. Each algorithm sums predictor scores to determine eligibility for AHI testing.

A recent pooled analysis of the data used to develop the four existing screening algorithms produced a ‘consensus’ AHI screening algorithm for use in young adults aged 18–35 years, based on seven characteristics: younger age (i.e. 18–29 years), reported fever, fatigue, body pains, diarrhoea, sore throat and genital ulcer disease (GUD).^17^ All characteristics except GUD receive a score of 1; GUD receives a score of 3. Patients who have a score ≥2 are eligible for AHI evaluation. A screening score of at least 2 would indicate AHI testing for 5–50% of participants in the data set in which this consensus screening score algorithm was developed, substantially decreasing the number needing testing. The performance expressed as the area under the receiver operating characteristics curve (AUC) for the algorithm was 0.78.^17^ Individuals with discordant rapid test results are at high risk for AEHI a priori.^20^ These individuals and those for whom AHI testing would be indicated, based on a risk score, require additional testing using either HIV-1 RNA or p24 antigen detection or repeat rapid HIV tests 2–3 weeks following the discordant test results.^21^

### Pilot testing and participant selection

Although the AHI algorithm has not yet been validated in other settings, the authors decided to train COs to use this screening algorithm for two main reasons:

- the yield of this algorithm has been assessed in the large sub-county hospital from which some of the COs for the present study were selected;^22^
- this is the only algorithm to exclusively use symptoms and age to derive a score.^12^

In Malindi, participants were mainly recruited from the outpatient department at which a study of AHI screening and testing was ongoing. The authors advertised the course during clinic hours and liaised with a senior CO who offered the course to their colleagues. Malindi sub-county hospital has 28 qualified COs and 21 intern COs rotating through various departments.

Two pilot test sessions were undertaken. The first included 17 COs working at the outpatient department of Malindi District Hospital, a coastal sub-county hospital. The second session was organized at a CO training school in Mtwapa and delivered to 28 final year students.

Each session consisted of obtaining formal consent, an introduction to the training provided by the training facilitator, a short demographic questionnaire, the pre-test questionnaire, the case study exercise (Table 1), reading of the module by each participant, the post-test questionnaire, the screening algorithm exercise, a review of the questionnaire answers, time for questions for the facilitators, formal feedback and, finally, certificate presentation. The screening algorithm exercise was developed for the second session, with the COs in training, after challenges with comprehension emerged in the first training session.

**Table 1.** Characteristics of 45 in- and pre-service clinical officers (CsO) selected for self-directed module in Malindi and Mtwapa, Kenya, 2016

Registration characteristics	All	In-service COs	Pre-service COs
	N = 45	N = 17	N = 28
	n (%)	n (%)	n (%)
Gender			
Male	35 (77.8)	9 (52.9)	26 (92.9)
Female	10 (22.2)	8 (47.1)	2 (7.1)
Nationality			
Kenya	17 (37.8)	0 (0.0)	17 (60.7)
Somali	11 (24.4)	0 (0.0)	11 (39.3)
Missing	17 (37.8)	17 (100.0)	0 (0.0)
Job department			
Outpatient department	8 (17.8)	8 (47.1)	0 (0.0)
Casualty	2 (4.4)	2 (11.8)	0 (0.0)
Paediatrics	3 (6.7)	3 (17.6)	0 (0.0)
Gynaecology	2 (4.4)	2 (11.8)	0 (0.0)
Comprehensive care centre	2 (4.4)	2 (11.8)	0 (0.0)
Training school	28 (62.2)	0 (0.0)	28 (100.0)
Clinical experience (years)			
1	36 (80.0)	8 (47.1)	28 (100.0)
2–5	4 (8.9)	4 (23.5)	0 (0.0)
5+	5 (11.1)	5 (29.4)	0 (0.0)

While there was no formal time limit for any section of the training session, reading of the training module lasted on average 45 minutes Each session was limited to 3 hours. There were at least two facilitators from KEMRI available at each session. They introduced the training session, explained the questionnaire answers at the end of the session and were available for questions throughout the session, including dedicated time at the end for questions. Each participant was given a study identification number (study ID), which was written at the top of the pre- and post-test questionnaire, the case study and the screening algorithm exercise, so each could be identified as belonging to a specific trainee. Both training sessions were conducted on Saturday mornings. Participants received a small reimbursement for their time (500 Ksh, or US$ 5).

### Pre- and post-tests

To evaluate the efficacy of the self-directed module, 20 pre- and post-test multiple-choice questions were designed. There were five questions for each domain as outlined above. All the questions were developed from information provided in the module (Supplementary information).

### Screening algorithm exercise

Participants from the first training session scored poorly on two questions in the ‘symptoms and screening algorithm’ domain. To improve the training materials, a new screening algorithm exercise was, therefore, designed for the second training session. In this exercise, participants were provided with the screening algorithm, and given 10 case studies, including patients with varying age and symptoms. They were asked to identify the patients who should be evaluated for AHI.

### Case study exercise

A case study exercise was included prior to the module to assess clinical competency and knowledge of current guidelines for managing patients presenting with a fever.^23^ This exercise described a young person with a fever and asked participants to form a differential diagnosis for this patient.

### Data analysis

Participants in the two pilot test sessions provided limited socio-demographic data (gender, years in service, country of origin, department currently working for if in service). AHI knowledge was assessed before and immediately after the training. Binary and categorical baseline demographic characteristics of study participants were assessed using χ^2^ tests. Median differences before and after the training module were compared using Wilcoxon signed rank test for matched pairs. Differences between paired proportions before and after training were compared using McNemar’s test. Differences in scores between two or more groups were compared using the Wilcoxon–Mann–Whitney test. Analysis was conducted using Stata 13.0 (StataCorp LP, College Station, TX, USA).

## Results

A total of 17 in-service COs and 28 pre-service COs took the self-directed training module. The majority of participants were male, and only a fifth reported having 2 or more years of work experience (Table 2). Out of 17 in-service COs, eight (47%) reported working in the outpatient department, where AHI patients are most likely to present with symptoms.

**Table 2.** Increase in acute HIV-1 infection (AHI) knowledge from pre- to post-test among 45 in-service and pre-service clinical officers in Malindi and Mtwapa, Kenya, 2016

	Clinical officers and pre-service clinical officers, n	Pre-test (baseline) median (%)	Post-test median (%)	Difference between pre- and post-test multiple-choice questions (%)		
				Median difference (%)	Interquartile range	p-Value (Wilcoxon)
All	45	35	75	+40	30–50	<0.001
Gender						
Male	35	35	75	+40	30–50	<0.001
Female	10	35	75	+35	20–50	0.005
Job title						
Clinical officer	17	40	75	+30	20–40	<0.001
Pre-service clinical officer	28	35	80	+45	35–55	<0.001
Domain						
Part 1 (q1–q5)		60	100	+20	0–40	<0.001
Part 2 (q6–q10)		40	80	+60	40–60	<0.001
Part 3 (q11–q15)		20	60	+40	20–60	<0.001
Screening algorithm (q14–q15)		0	0	0	0–50	0.170
Part 4 (q16–q20)		40	80	+40	40–60	<0.001

Table 3 shows the change in AHI knowledge from pre- to post-test assessments among 45 in-service and pre-service COs. Pre-test scores were low (median 35%, interquartile range [IQR] 30–45%). Scores significantly improved following training, with a median post-test score of 75% (IQR 70–85%), and median score improvement of 40% (IQR 30–50%, Wilcoxon signed-rank test p<0.0001). There was a borderline significant difference (40% vs 35%, Wilcoxon–Mann–Whitney test p=0.06) in the pre-test scores between in-service and pre-service COs. Pre-service COs had higher post-test scores than in-service COs (median improvement: 45% vs 30%, Wilcoxon signed-rank test p<0.001).

**Table 3.** Differential diagnosis of a febrile adult patient by clinical officers or pre-service clinical officers, Coastal Kenya, 2016

Differential diagnosis	All	Clinical officer (n=17)		Pre-service clinical officer (n=28)
	n (%)	n (%)		n (%)
Malaria	45 (100.0)	17 (100.0)		28 (100.0)
Acute HIV infection (AHI)*	32 (71.1)	9 (52.9)		23 (82.1)
Three or more differential diagnoses†*	36 (80.0)	11 (64.7)		25 (89.3)

*p-value ≤0.05.

†Including meningitis, typhoid fever, bacteraemia/sepsis/bacterial infection, respiratory tract infection (including rhinitis, tonsillitis and pharyngitis), acute viral infection, urinary tract infection, dengue fever, rheumatic fever, sexually transmitted infection, myalgia, brucellosis, gastroenteritis, otitis media.

Despite large improvements in the pre- and post-test scores in all four domains, there was no improvement in responses to the two questions that concerned the utility of the screening algorithm (median increase 0%, IQR 0–50%, Wilcoxon signed-rank test p = 0.17). The questions were numbers 14 and 15: ‘Why is a screening scoring system helpful?’ and ‘Which of these patients should you send for AHI testing (only one)?’ (Supplementary information: questions 14 and 15).

### Screening algorithm exercise

Out of 28 pre-service COs who completed the screening algorithm exercise, the median score was 7 (IQR, 5–7) out of a score of 10. Only one pre-service CO correctly scored all 10 cases.

### Case study

All participants suggested malaria as a differential diagnosis for a young adult with a fever, in line with WHO guidelines. A total of 52.9% of in-service COs listed AHI as a differential diagnosis compared with 82.1% of pre-service COs (p<0.05) (see Table 1).

Overall, a higher proportion of pre-service COs than in-service COs listed three or more differential diagnoses for the case presented (89% vs 65%, p=0.046). There was no correlation between a higher number of differential diagnoses and correct scoring of the two questions concerning the screening algorithm or correct scoring of the 10 questions in the screening algorithm exercise (data not shown).

## Discussion

COs involved in this study had poor baseline knowledge of AHI, which is not surprising given that AHI is not included in the curriculum training for COs in sSA. While real-time AHI diagnosis would require the availability of advanced diagnostic assays (e.g. p24 antigen or HIV-1 RNA testing), repeat rapid antibody testing for patients reporting symptoms compatible with AHI would allow for the diagnosis of AEHI in most resource-limited settings.^21^ However, the lack of clinical guidelines on AHI diagnosis and testing in sSA also means that in-service COs will not test for or treat AHI.^11^ This means they will not develop their knowledge through clinical practice as they might do with conditions they commonly diagnose and treat.

AHI knowledge significantly improved following reading a self-directed module, prompting us to revise and improve the AHI training module for online access, in order to reach more trainees. This would allow a wider dissemination of AHI knowledge to anyone with an Internet-connected device. The module has since been converted into an online version and is freely accessible on www.marps-africa.org. The content is similar to the paper version and some of the figures have been made interactive to improve the interface for users. The online version includes the pre- and post-test questionnaires, along with the screening algorithm exercise. In addition, demographic information about trainees, along with pre- and post-test scores, are collected. Tests can be retaken after the initial attempt and the scores updated. The aim was to record the first attempt scores for both pre- and post-test questionnaires to help us further evaluate the effectiveness of the module.

Correctly identifying patients for AHI evaluation through the screening algorithm was the biggest challenge to implementing screening in a clinical context. Given that participants were provided with the algorithm, it was expected that the majority would correctly identify all patients needing AHI testing. One potential confusion with application of the algorithm is the score given for being in the 18–29 age bracket. Participants seemed to confuse this with the fact that the algorithm was developed for patients aged 18–35 years;^17^ they did not understand giving 1 point to younger people within this larger age bracket. In the revised online version of the module, the age bracket for AHI screening was expanded to include adults aged 18–39 years, as HIV incidence remains high among Kenyans in this age range.^24^ This addition helped to make a clearer distinction between the age group 18–29 years, worth 1 point in the algorithm, and the age group 30–39, not awarded any points. In the online AHI screening algorithm exercise consisting of 10 cases, an immediate response after each answer is chosen was provided, allowing for each case question to be taken again if the first answer was incorrect. Evaluation of data from the online curriculum will be used to determine whether these changes have improved understanding of the screening algorithm.

All clinicians suggested malaria as a cause of fever, in concordance with WHO and National testing guidelines.^23^ Only 71.1% of participants volunteered AHI as a cause of fever. Considering the event was advertised as an AHI training session this probably reflects COs’ poor knowledge prior to the module. The number of differential diagnoses varied greatly between participants. Pre-service COs volunteered significantly more differentials than in-service COs. This may be due to increased or more up-to-date knowledge, or it could be that they are more used to examinations and, therefore, will volunteer more answers.

While discussion about AHI has been limited in most HIV prevention and care guidelines in sSA until recently, a glimmer of hope is emerging through the recent roll out of pre-exposure prophylaxis (PrEP) targeting populations at substantial risk of HIV acquisition.^19^ Guidelines in Kenya and elsewhere discuss the importance of recognition of AHI symptoms, and provide guidance to clinicians to postpone PrEP when patients present with symptoms compatible with AHI.^19^ Greater availability of point of care HIV-1 RNA testing would allow providers to diagnosis AHI earlier when symptoms are present. Currently, providers are advised to retest such patients 1 month after the last negative HIV test before reconsideration for PrEP.

While a laptop or desktop computer may not be available in all health facilities, mobile phone usage is currently increasing throughout sSA: 41% of sub-Saharan Africans were estimated to have access to a mobile phone in 2015, with 51% predicted to have mobile broadband access by 2020 (GSMA, The Mobile Economy 2015). To make use of this valuable resource, it may be beneficial to develop an application of the online training and the AHI screening algorithm for mobile phones, in order to make these resources more accessible to clinicians. If the screening algorithm were provided in an interactive format, allowing providers to input patients’ age and symptoms, and generate a score, the application could advise whether to test for AHI and describe options for testing.

WHO guidelines now recommend starting ART as early as possible after HIV diagnosis, including for patients diagnosed during AHI.^25^ New treatment guidelines and increasing availability of test platforms (i.e. Xpert machines) for AHI diagnosis mean that AHI can now be diagnosed and treatment initiated without untoward delays. The impact of an HIV-1 RNA testing intervention to identify undiagnosed acute and prevalent HIV infection in Kenyan health facilities is currently being tested in a 2875-person proof-of-concept study funded by the US National Institutes of Health (R01AI124968, co-PI Sanders and Graham). In order to halt ongoing HIV transmission anywhere in the world, patients with AHI should be identified as soon as possible. For AHI diagnosis to become part of clinical practice, adequate training of clinical staff is required. Although the online AHI module may be helpful, the development of specific guidelines to address AHI diagnosis and treatment is needed.

## Conclusion

AHI is a common and important condition of which clinical officers in Coastal Kenya have poor knowledge. A self-directed learning module was successful at improving knowledge about AHI; however, the cohort struggled to utilize the screening algorithm. The pilot-tested module has been revised and adapted for online delivery, and further evaluations and refinement are planned. Clinical knowledge of AHI is key to improving diagnosis and management. While training to increase knowledge about AHI is important, specific guidelines are urgently needed to address AHI diagnosis and management.

## 

**Author’s contributions:** HR and ES conceived the study. HR, ES, SG, RB and EV contributed to the module material. HR, ES, OC, CO, CG and EV facilitated the training sessions. HR and EW analysed the data, and the data were interpreted by ES and HR. HR and ES drafted the manuscript. ES, SG and SRJ critically revised the manuscript. All authors read and approved the final manuscript.

**Acknowledgments:** Many thanks to Mary-Anne Barckhoff for preparing the AHI module for online learning at www.maprs-africa.org., and the clinical officers from Malindi sub-County Hospital and the clinical officers in training from Mtwapa Training College for their participation and feedback on the module. Thanks also go to Dr Marianne Darwinkel, from Mtwapa Training school, for helping to organize the training session.

**Funding:** The Kenya Wellcome Trust Research Programme (KWTRP) at the Centre for Geographical Medicine Research-Kilifi is supported by core funding from the Wellcome Trust [#203077/Z/16/Z]. This work was partially funded by International AIDS Vaccine Initiative (IAVI) with the generous support of USAID and other donors; a full list of IAVI donors is available at www.iavi.org. EJS receives research funding from IAVI, the National Institute of Health (NIH) [grant 1R01AI124968] and the Wellcome Trust. SMG was supported by NIH grant R01AI124968. This work was also supported through the Sub-Saharan African Network for TB/HIV Research Excellence (SANTHE), a DELTAS Africa Initiative [grant # DEL-15-006]. The contents are the responsibility of the study authors and do not necessarily reflect the views of USAID, the NIH, the United States Government or the Wellcome Trust. This report was published with permission from KEMRI.

**Competing interest:** None declared.

**Ethical approval:** Approved by Science and Ethical Review Unit of KEMRI.

Presented in part at HIVR4P conference, Chicago, Illinois, USA, 17–21 October 2016 in poster format (P09-10)

## Supplementary Material

Supplementary Data
