# Supplementary material for: Pilot testing of an online training module about screening for acute HIV infection in adult patients seeking urgent healthcare
Source: Int Health. 2018 Nov 2;11(2):93–100. doi: 10.1093/inthealth/ihy077 (PMC6398591; doi:10.1093/inthealth/ihy077)
Supplement: Supplementary Data [file ihy077_supplementary_information.docx]

**<CT/>Pre-/Post-test Questionnaire</CT>**

Test number:______

Please complete the following single best answer questions to the best of your knowledge. Please give only one answer per question:

1. What is acute HIV infection (AHI)?
   1. The 1st year after HIV infection.
   2. The time the HIV RNA test is positive, but the rapid Ab test is negative.
   3. The time the rapid Ab test is positive, but the HIV RNA test is negative.
   4. The time the rapid Ab test and the HIV RNA test are positive.
2. What is prevalent HIV infection?
   1. Early and chronic HIV infection, when the rapid Ab test is positive.
   2. The time when the viral load has stabilized.
   3. The time after symptoms have developed.
   4. The period 6 months before AIDS develops.
3. Which of the following statements concerning AHI is not correct?
   1. AHI is a highly infectious period of HIV.
   2. Symptoms may develop during AHI.
   3. Patients often attend healthcare facilities during AHI.
   4. AHI lasts for several months.
4. Why is acute HIV important to diagnose?
   1. Earlier treatment is better for the patient.
   2. Risk reduction counselling can be given to prevent onward transmission.
   3. Patients who start on ART soon after diagnosis will reduce their viral load and ability to transmit the virus.
   4. All of the above.
5. Why is earlier treatment better for patients?
   1. To reduce the need for expensive CD4 counts.
   2. To reduce side effects.
   3. To improve the patient’s prognosis by preserving immune system function.
   4. All of the above.
6. What happens to the viral load during acute HIV infection?
   1. It slowly increases.
   2. It dramatically increases.
   3. It stays the same.
   4. It decreases.
7. Which HIV tests can be used to diagnose AHI?
   1. HIV RNA and HIV rapid test.
   2. HIV p24 and HIV rapid test.
   3. HIV RNA and HIV p24.
   4. Only the HIV rapid test.
8. What is the first HIV test to become positive after HIV is acquired?
   1. HIV RNA.
   2. HIV rapid Ab test.
   3. HIV p24.
   4. HIV p67.
9. How long after HIV infection does the rapid HIV test become positive?
   1. Immediately (day 1).
   2. Day 7.
   3. Day 21.
   4. Day 45.
10. What do discordant rapid tests suggest?
    1. Poor testing kits.
    2. Possible AHI or early HIV infection.
    3. Test carried out incorrectly.
    4. Any of the above.
11. What percentage of people experience symptoms during AHI?
    1. 5–15%.
    2. 10–60%.
    3. 40–90%.
    4. 100%.
12. Which of the following symptoms are not common in AHI?
    1. Sore throat.
    2. Fever.
    3. Headache.
    4. Cough.
13. When do symptoms usually develop following HIV acquisition?
    1. The day after infection.
    2. 1 week after infection.
    3. 2 weeks after infection.
    4. 1 month after infection.
14. Why is a screening scoring system helpful?
    1. It helps the clinician decide who needs a test.
    2. It means fewer people need the test.
    3. It prevents missing most cases of AHI.
    4. All of the above.
15. Which of these patients should you send for AHI testing (only one)?
    1. Michael, a 19-year-old with a sore throat.
    2. Fatima, a 23-year-old with a cough.
    3. Clement, a 35-year-old with a fever.
    4. Thomas, a 45-year-old with fever and sore throat.
16. What do WHO guidelines recommend for AHI testing?
    1. No recommendations.
    2. Test all adults with an observed fever.
    3. Test all adults with a reported fever.
    4. Test all adults presenting for urgent care.
17. When do new WHO guidelines recommend commencing ART?
    1. As soon as possible after diagnosis.
    2. When the CD4 count drops below 500.
    3. When the CD4 count drops below 350.
    4. When infections start to develop.
18. How can AHI be treated?
    1. Early ART.
    2. Symptomatic management.
    3. Hospitalization for severe symptoms.
    4. All of the above.
19. How long after care seeking for potential AHI symptoms should a rapid HIV test be repeated if indicated?
    1. 1–2 weeks.
    2. 2–3 weeks.
    3. 1–2 months.
    4. 2–3 months.
20. In discussion about AHI testing with a patient, the following topics should be covered:
    1. AHI is possible, but unlikely.
    2. AHI is possible, but unlikely and treatment is available.
    3. AHI is possible, but unlikely, treatment is available and partner(s) should attend for testing.
    4. AHI is possible, but unlikely, treatment is available and partner(s) do not need to attend for testing.

**<H1/>Case study exercise</H1>**

Test number:______

John is 22. He has come to the outpatient department reporting a fever and has been feeling generally unwell for the last 2 days. No specific symptoms are found on further questioning. On examination his temperature is 38.2⁰C, with no other findings.

1. In addition to malaria, what other diagnoses would you consider?
2. What investigations would you order?

**<H1/>Screening score exercise</H1>**

Test number:_____

Here is the screening score algorithm:


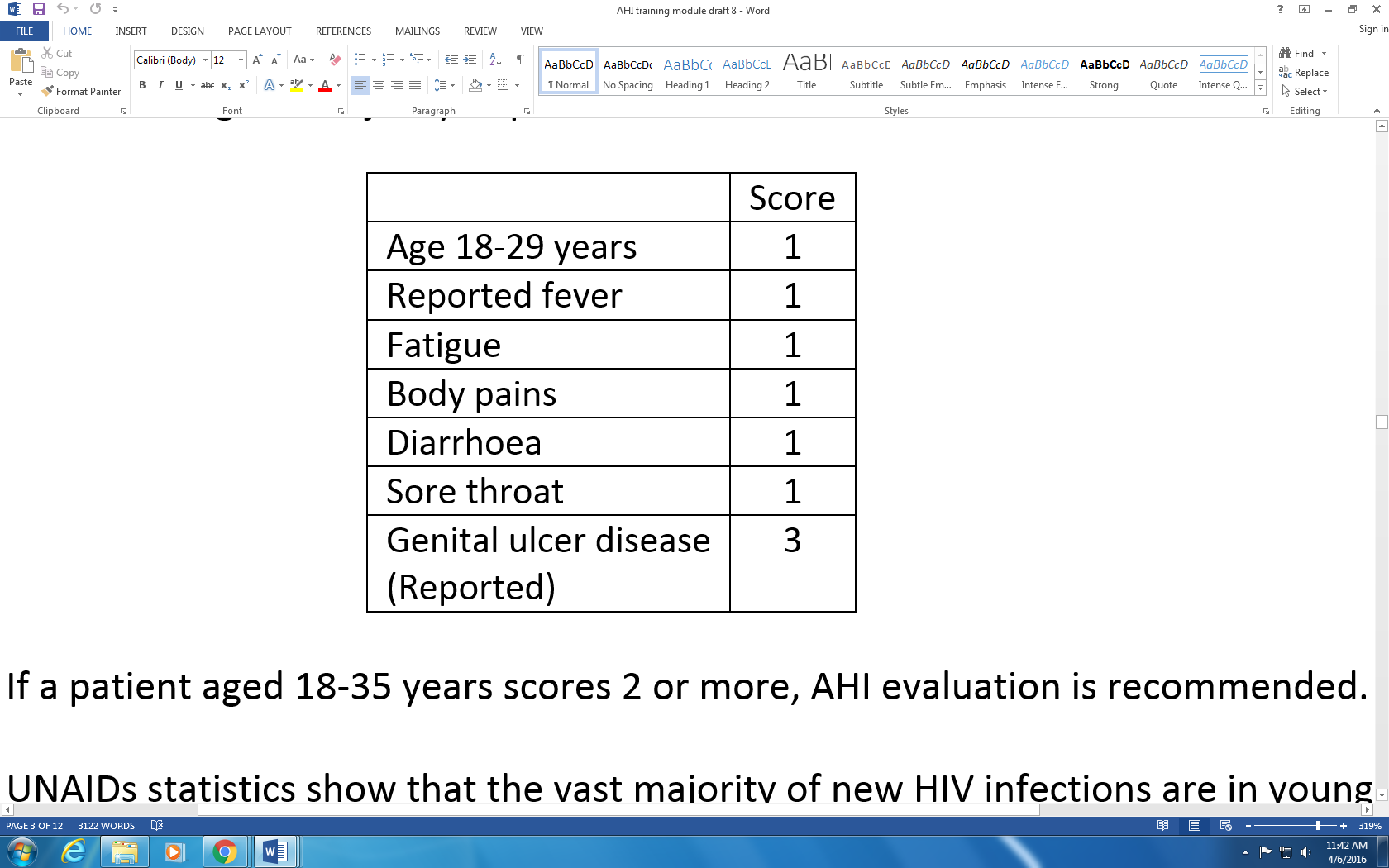


Which of these patients should be screened for AHI (circle the correct answers)?

1. A 19-year-old with a sore throat.
2. A 45-year-old with body pains, fatigue, diarrhoea and fever.
3. A 17-year-old with fever and fatigue.
4. A 28-year-old with cough and body pain.
5. A 30-year-old with diarrhoea and fatigue.
6. A 35-year-old with a fever and a cough.
7. A 34-year-old with genital ulcers.
8. A 31-year-old with fever.
9. A 37-year-old with fevers, body pains and a sore throat.
10. An 18-year-old with fatigue.
